# Description of Twenty-Nine Animal Hoarding Cases in Italy: The Impact on Animal Welfare

**DOI:** 10.3390/ani13182968

**Published:** 2023-09-20

**Authors:** Luigi Sacchettino, Claudia Gatta, Viviana Orsola Giuliano, Francesca Bellini, Alessia Liverini, Francesca Ciani, Luigi Avallone, Danila d’Angelo, Francesco Napolitano

**Affiliations:** 1 Department of Veterinary Medicine and Animal Production, University of Naples Federico II, 80137 Naples, Italy; luigi.sacchettino@unina.it (L.S.); claudia.gatta@unina.it (C.G.); ciani@unina.it (F.C.); avallone@unina.it (L.A.); 2 Veterinary Behaviorist, 81100 Caserta, Italy; vetvivianagiuliano@gmail.com; 3 Local Health Unit Rome 1, 00193 Rome, Italy; francesca.bellini@aslroma1.it; 4 Local Health Unit Rome 4, 00053 Rome, Italy; alessia.liverini@aslroma4.it; 5 CEINGE-Biotecnologie Avanzate Franco Salvatore, 80145 Naples, Italy

**Keywords:** animal welfare, human animal relationship, animal behavior, hoarding disorder, hoarding, compulsive behavior

## Abstract

**Simple Summary:**

Animal hoarding is a human psychiatric disease, characterized by a compulsive collection of animals, which generally produces a deep suffering for both animals involved and hoarders themselves. Here, we sought to analyze and profile 29 animal hoarders, who lived within urban and rural areas of the Lazio region (Italy), according to sex, age, job, living conditions and reasons of the patients behind such a pathological disorder. We also outlined the number and different animal species for each case analyzed. The animal hoarding phenomenon severely impacts human health, animal welfare and the environment worldwide, thus calling into question thoughtful strategies to be implemented, aimed at better coping with such a social issue. In this respect, the establishment of an animal hoarding observatory at a national level able to coordinate actions to be jointly taken might be encouraged, to establish an effective and adequate strategy to recognize this phenomenon, and to safeguard animal health.

**Abstract:**

The hoarding of animals is a psychiatric disease, characterized by a compulsive collection of animals, with a relevant impact upon the care and welfare of animals, as well as on human society. In Italy, there are neither substantial reports nor information shared about such a phenomenon, making it difficult to draw a clear picture of the hoarder profile. Therefore, in the present work, we sought to detail 29 cases of animal accumulators in Italy, who lived within two areas of the Lazio region, and accumulated a total of 1080 animals from 2019 to 2022. In line with other international studies, we observed a prevalence of middle-aged (in their fifties) women, who lived mainly alone in a high level of social and health degradation. Most of the hoarded animals exhibited severe signs of dehydration and malnutrition, muscle hypotrophy, dermatological injuries, and behavioral disorders. Animal hoarding is not yet fully understood nor recognized as a psychosocial disorder, although it produces a deep suffering for the hoarder themselves, as well as corresponding family members, and the animals accumulated. Therefore, given the crucial impact of animal hoarding upon human and animal welfare, cross-cultural networks aimed at properly raising awareness of the problem could be established.

## 1. Introduction

Animal hoarding or the “compulsive hoarding of animals” is a psychiatric disease with a significant impact on both animal welfare and society. According to the Diagnostic and Statistical Manual (DSM)’s fourth updated edition [1], symptoms of the hoarding disorder might be associated with obsessive compulsive disorder (OCD) or obsessive compulsive personality disorder (OCPD). OCD is characterized by intrusive images, thoughts or obsessions that the patient tries to avoid by engaging in behaviors (compulsions) meant to suppress the intrusion and offer relief from it [2]. Considering the greater attention toward such a disorder, the fifth version of the DSM reports a specific chapter to the accumulation disorder, “Hoarding Disorder” (HD), which is included in the new section, “OCD and Related Disorders” [3]. Based on the study of Williams (2014), the hoarder is incapable of offering even the minimum care of animals, as well as recognizing the suffering state they are experiencing, thus turning into an uncontrolled hoarding of both animals and objects in general, and leaving environmental conditions neglected [4].

Due to the overwhelming number of household animals accumulated, mainly cats and dogs, the situation is for the most part one of confinement in small and inadequate places [5]. These animals are often hungry, caged, stacked and even dead. As a result, animal hoarding disorder is a complex phenomenon and has a severe impact on both the legal system and public health [4,5,6,7,8]. The increasing number of published case reports worldwide highlights a great interest from the scientific community about this topic [9,10,11,12,13,14], pointing toward a more accurate interdisciplinary approach, to the early identification of potential hoarders and to improve the quality of both patients and animals who live with them; moreover, the extremely high recent documented recurrence rates demonstrates the ineffectiveness of the present management strategies [5,6,8]. Sociodemographic factors such as gender, age, family arrangement, educational attainment and previous interaction with hoarders also come into play for the recognition of the problem [15,16,17]. Recent data reported that animal hoarders are usually women and elderly individuals who, more often, live alone. Nevertheless, a low educational level does not tightly correlate with disorder severity level, since contrasting data were recently reported by Stumpf et al. [18]. In line with the review by Paloski and colleagues, hoarders may also experience co-occurring psychotic symptoms, including delusional thinking, based on the strong belief of their special attitude to empathize with animals and care for them flawlessly, ignoring the obvious distressing conditions [8]. In Italy, there have been no reports and exchanges of information on this phenomenon and on the animals involved, and there has been only a small number of reported cases. In this regard, there is currently only one research in Italy [19], which describes the health, legal and veterinary aspects of one animal hoarding case, that has remained unsolved, of a woman suffering from animal hoarding, which emerged in 2005. Therefore, herein, we have collected information about 29 cases of animal accumulators in two areas of the Lazio region (Italy), involving 1080 animals during 2019–2022, to obtain useful data to identify the profile of the hoarder, safeguard the animal welfare and sensitize the scientific community, in the perspective of “One-Health”.

## 2. Materials and Methods

**Figure 1:**
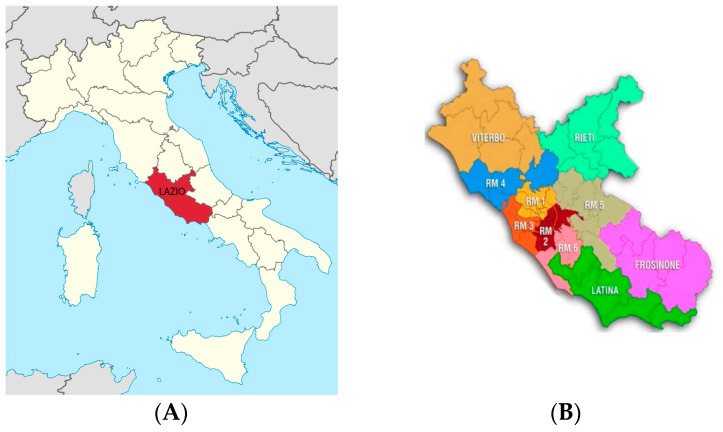
Geographical representation of Italy: in red, the Lazio region, situated in the central part of the country (**A**). Representation of Lazio region with the distribution of regional ASL: in orange, ASL Rome 1; and in blue, ASL Rome 4 (**B**).

In the present study, we have analyzed the data collected from specific areas of the Lazio region (Figure 1A) situated in the central peninsular section of the country, with around 5,714,882 inhabitants. This work had arisen from the collaboration between the Department of Veterinary Medicine and Animal Production, University of Naples—Federico II, and the Local Health Units (ASL) 1 and 4 of Rome (Lazio region), with the aim to emphasize the problems related to animal welfare and to trace a profile of the hoarder during the period 2019–2022.

Twenty-nine cases of hoarders were investigated in relation to a total of 1080 animals involved. The data were collected from records compiled by veterinarians of ASL (Figure 1B) during supervisions carried out, following reports from different sources (firefighters, policemen, zoophile guards and neighbors) who reported to the ASL.

For the acquisition of data, we used a modified form of that by Ascione, 2010 [20], aimed at collecting useful information to outline the demographic data of hoarders, the sanitary conditions of their houses and the animals involved. The data were collected on the forms directly by veterinarians attending cases.

The form consisted of 10 items divided into the following sections: demographic data of the hoarder (age, gender, job, family group composition, marital status, residence type); motivation provided by the hoarder; stakeholders involved in the cases; degree of environmental neglect; number, type, clinical and behavioral signs of all accumulated animals; and case resolution (see Supplementary Materials).

### Statistics

Statistical analysis was performed using GraphPad Prism Version 10.0.2.

## 3. Results

### 3.1. Animal Hoarding Social Demographic Data

**Figure 2:**
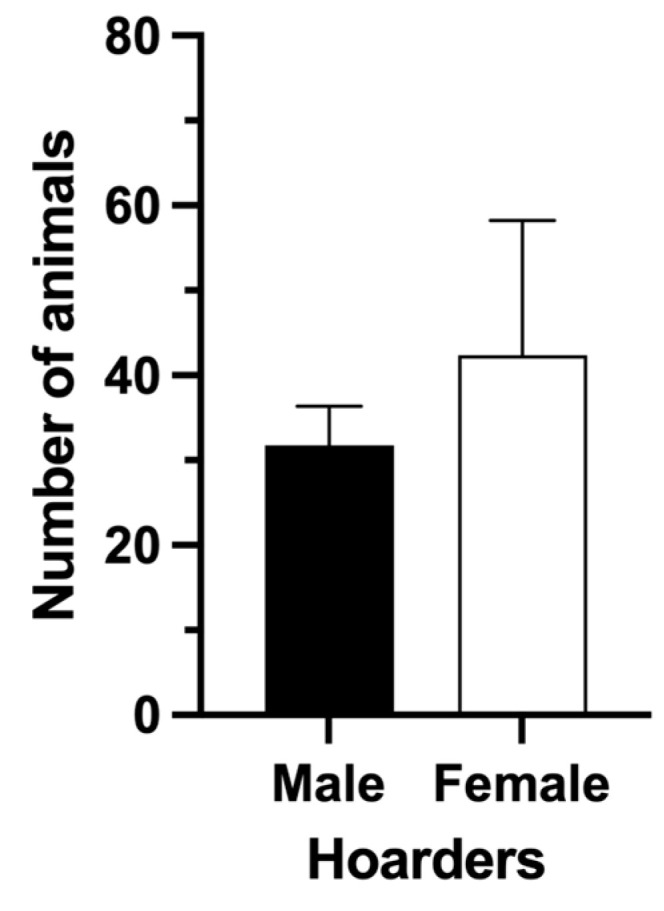
Hoarder gender vs. animals involved. The gender of the hoarder has no statistical impact on the number of animals involved.

The 29 cases involved individuals divided by gender: 52% were women and 48% were men. The number of the involved animals was not affected by the hoarder gender (*p* = 0.3046, Mann–Whitney test, Figure 2).

The average age of the entire hoarder sample is between 50 and 60 years (35% and 31%, respectively). The majority of cases concerned people living alone (52%), while 28% were divorced and widowed people and only 20% married or cohabiting. The profile of hoarders analyzed showed the type of job, which varied: doctors, nurses, teachers, freelancers, employees, unemployed and pensioners. Alongside pensioners, almost 50% of hoarders were employed as health workers, teachers, freelancers and in private practices (Table 1).

### 3.2. Degree of Environment Neglect and Motivation

In the observed cases, 52% were people living in apartments with severe conditions of neglect, very often incompatible with life, characterized by a high accumulation of objects (i.e., inability to access some rooms, given the degree of neglect), food leftovers, a lack of water and electricity, unsanitary conditions, and the presence of excrement. Additionally, 41% lived in an environment with a high accumulation of objects, a lack of residential potential and few usable services. In contrast, only 7% of hoarders showed a moderate degree of neglect, with a portion of their home services still usable and only a few accumulated objects. The observed accumulators tended to justify their behavior: in 45% of cases, they declared that they wanted to save animals (“rescue hoarder”), 24% felt a love for animals, 7% considered them the only friends they had, 7% considered them as children, while 17% justified their actions with other reasons.

### 3.3. Animals Involved and Health Conditions

**Figure 3:**
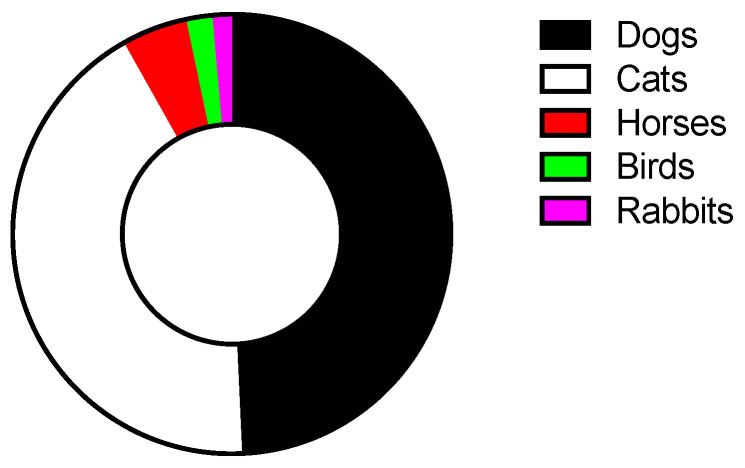
Animals involved in this research; the animal species most targeted by animal hoarding were dogs and cats.

The information on the number of animals and the percentages of clinical and behavioral problems described herein are taken from the reports drawn up by the veterinarian responsible for the local animal health holding present during the supervisions. The most common animal species involved were dogs (*n* = 532), closely followed by cats (*n* = 460), horses (*n* = 52), birds (*n* = 21), and rabbits (*n* = 15) as revealed by the overall significant animal species effect (chi-squared test, *p* < 0.0001) (Figure 3).

The signs and symptoms found during general physical examinations were grouped into three categories: (1) animals with appropriate health conditions, (2) animals with severe health conditions and (3) dead animals. The 67% (*n* = 723) of animals involved showed severe conditions of health, characterized by dehydration, serious malnutrition, skin lesions (i.e., bite injuries and alopecies spread all over the body) and muscle atrophy. In some, there were also deviations of the spinal column.

The 32% (*n* = 346) of the affected animals showed moderate malnutrition, mild dermatitis and superficial skin lesions (localized alopecia of elbow, hock, pelvis).

Some animals showed signs of behavioral discomfort or insecurity toward humans, in particular, such animals were wary of human manipulation, fearful of noise or sudden gestures and presented stereotypes such as licking walls and tail chasing.

Unfortunately, 1% (*n* = 11) were found dead, inside cages, washing machines, refrigerators or found in the gardens of the respective houses.

### 3.4. Distribution of Cases by Area

The comparison of cases was carried out in two distinct areas of the Lazio region: ASL Rome 1, classified as an urban area; and ASL Rome 4, classified as a rural area. In the urban area, 49% (*n* = 529) of animals were involved, of which 69% (*n* = 365) were cats, 24% (*n* = 128) dogs, 4% (*n* = 21) birds and 3% (*n* = 15) rabbits. In the rural area, 51% (*n* = 551) of animals were involved, including 73% (*n* = 404) of dogs, 17% (*n* = 95) of cats and 10% (*n* = 52) of horses.

### 3.5. Resolution of Cases

The resolution of several cases was the seizure of all animals (38%; *n* = 11); in 7% (*n* = 2) out of 29 cases, the accumulators were subjected to psychiatric examination/mandatory medical treatment; in 17% of cases (*n* = 5), the accumulators were submitted to legal procedures; and in 38% of cases (*n* = 11), the phenomenon remained unresolved.

## 4. Discussion

In the present retrospective examination, we described 29 animal hoarding cases occurring in Italy during 2019–2022, with the aim of focusing the welfare status of the animals in terms of behavioral aspects of animals targeted in such a psychiatric disorder. In line with a previous Spanish study by Calvo et. al., we reported a slight increase in female hoarders (52%) [11]. However, other studies performed reported a clear predominance of females, exceeding 60%, most likely because they reviewed more cases [12,14,21]. According to Mathes et al. (2019), gender may affect the onset, appearance and severity of OCD symptoms since females are more likely to develop OCD between adolescence and adulthood [22]. However, further studies on a wider cohort of patients need to address this issue. Most of the hoarders in our sample were middle-aged (in their fifties and sixties), which agrees with previous international reports [14,21,23,24]. Accordingly, Paloski and colleagues [8] hypothesized a correlation between animal hoarding occurrence and aging, which is sometimes regarded as a trigger or consequence of the undiagnosed neurological and/or psychiatric disorders. However, deeper studies about such topics have to be carried out. We reported that the highest percentage of the hoarders consisted of people living alone, hinting at the potential impact of loneliness on the genesis of the disorder [14,21]. Animals represent the focus of attempts to repair disappointments and failures in life relationships or social isolation, having the ability to provide “emotional comfort” [25,26,27]. Animal hoarding generally occurs regardless of cultural and economic status [28,29]. Accordingly, we found that 45% of the patients had a high educational level, so they were teachers, health workers, freelancers and employed in private practices.

In a complex interplay with biological predispositions, contextual, social, and cultural factors, as well as individual characteristics and experiences, influence both human-directed and animal-directed empathy [18]. For instance, some psychological characteristics of humans, such as a desire for power and anger, are adversely correlated with animal empathy. While hostility temporarily reduces empathy, increases aggression, and lessens sensitivity to animal suffering and mistreatment, the need for power leads to a utilitarian view of people and animals as tools for self-gratification rather than living beings, who deserve respect and concern [18]. The hoarders present several reasons to explain and justify their accumulation of animals, denying all the allegations and claiming that the animals are well cared for, since they are the only ones who can save them or love them as if they were children [8,18,23,27]. In the present study, we noticed that the patients firmly believed that they were in charge of saving animals (45%) and considered themselves the only qualified people capable of satisfying the animal needs in the best way ever [21]. Our findings are supported by Arluke et al., who reported that animal hoarders frequently experienced an overwhelming urge to acquire animals to prevent negative events from happening to them [30]. Patients generally ignore either pain or low quality of animal care, since they deem to be right [19,23], so that they may experience intense agony when authorities attempt to remove them from animals (or objects) [30]. People who suffer from animal hoarding likely fall in the dissociative process that provide them with the illusion of living in a “parallel” yet unreal world and, differently to what is often observed for object hoarders, appear less prone to undergo therapeutic treatments [29]. We found a severe environmental disorder in the cases observed, including a high accumulation of objects, food leftovers, the lack of water and power, and the presence of droppings. This is not surprising, since the accumulation disorder makes the dwelling inadequate and not functional, with a high risk of zoonotic infections and disorders, for both visitors and neighbors [24,27,31].

Most of the animals involved in our work were dogs (49%) and cats (43%), in line with what was previously described [9,11,14]. From our perspective, the higher percentage of dogs involved can be also traced back to the role of experiences, culture in human–animal relationships and attachment to dogs as well [30,32], although the situation may be different depending on the location taken into consideration [33]. People generally do not see all animals as equal, but their physical and behavioral traits play a fundamental role in the way they are perceived, considered and treated [34]. In fact, they strive to prefer animals phylogenetically close to them, and show greater empathy and concern for them, neglecting to provide them the necessities they need [8]. Indeed, published data well described the detrimental conditions of animals hoarded, thus mirroring what we described in the present report [9,10,19]. In particular, many of the animals analyzed showed compromised health conditions, with evident clinical and behavioral issues. Some of them were even found dead and stored in the apartments, whereas others were dry, malnourished and cachectic, displaying widespread alopecies and dermatitis. In some cases, the animals had problems of muscular atrophy, due to the forced immobility, and a deviation of the spine caused by over-confinement in small cages.

In addition, they showed skin lesions caused by aggressions from other subjects (due to overcrowding, extremely poor social distancing and failure to satisfy basic ethological needs), contact with rigid surfaces (at bony prominence level) and compulsive licking, which is considered a coping strategy [35,36]. The hoarded animals showed a dysfunctional behavioral profile related to social fear as well as inter- and intra-aggression caused by a lack of necessary stimulation and improper socialization, which thus turns into a relevant increase in behavioral disorders, such as abnormal repetitive behaviors, excessive interspecies aggression, fear and anxiety [37,38]. Similar to humans, mistreated animals display anxiety, learned helplessness and hostility that severely compromise animal clinical and behavioral welfare [20].

Dead animals were only present in a few cases, in line with Spanish findings [11], but in contrast to previous data from Patronek in 1999 [23], who reported 80% of dead animals, which points toward the highly dynamic and diversified nature of the animal hoarding phenomenon.

This study found an uneven distribution of cases in the two areas described. In fact, most of the cases (68%) were reported in the urban area, Local Health Unit 1 of Rome, compared to the rural area of the Local Health Unit 4. In the urban area, the animals involved were mainly cats, followed by dogs, birds and rabbits; while in the rural area, there was a higher accumulation of dogs, followed by cats and horses. The increasing accumulation of cats in urban areas may be justified by the ease of keeping them in smaller spaces than dogs or other animals. The higher percentage of warnings in an urban context can be related to a higher population density and the presence of neighbors, who were annoyed by bad smells or loud noises.

Despite this phenomenon being considered as a psychiatric disorder, the animal hoarders cannot escape criminal liability [39]. In Italy, the article 544-ter of the penal code punishes anyone, who *«for cruelty or without necessity causes damage to an animal or subjects it to torture or to behavior or fatigue or unbearable work for its ethological characteristics […]»*, therefore condemning the contemptible treatment of animals, even if those responsible are in such conditions. Often, there is no evidence of intention. The Court of Cassation has clarified that, to resolve the crime, there need not necessarily be physical injuries present, and the suffering of the animals is sufficient; the law protects them as living beings that are able to perceive pain, even in the case of environmental and behavioral injuries (Cass. No. 46291/2003; Trib. Pen. To-Rino 25 October 2006). In Italy, therefore, an adult can choose to live in degraded situations without suffering any censorship. There is no law on public health or safety, but the act of keeping animals in poor hygienic conditions, leaving them without water or food, keeping them locked in narrow spaces and dirty with feces and depriving them of the possibility of movement is criminally punishable. As a psychiatric condition with a high risk of recurrence, it is essential to establish a specific support intervention and psychiatric rehabilitation therapy [40,41]. The possible solution of accumulation cases must include a calculation of the social impact in addition to the economic impact; in fact, the costs are attributable to the capture of animals, to their hospitalization for the detention and/or care in appropriate facilities and from the clearing of the apartment with environmental remediation operations (including disinfection and deratization).

Some limitations of the present study, including the small sample size, the small number of cases and difficulty in collecting data require further investigations by considering a wider geographic area, as well as more hoarding cases. Moreover, we struggled to acquire information from the hoarders, who generally appeared less cooperative with researchers and local health units. Taken together, this first and preliminary report about animal hoarding in Italy might pave the way for the generation of a standardized protocol to analyze and share data, from all Italian regions, aimed at creating a national observatory for the animal hoarding phenomenon.

## 5. Conclusions

We documented for the first time the hoarding disorder in two small areas of the Lazio region during 2019–2022. In particular, we profiled 29 hoarding cases, wherein those accountable had collected a total of 1080 animals, ranging from dogs and cats to horses, rabbits and birds. Overall, we highlighted the poor health conditions where the hoarded animals lived, with detrimental consequences upon human and animal welfare. Taken together, we suggest the development of a multidisciplinary approach, based on different job skills, along with the creation of common protocols to manage the animal hoarding phenomenon, aimed at safeguarding animal health and welfare.

## Figures and Tables

**Table 1:** Animal hoarding social demographic data, in percentage.

Gender	Age	Marital Status	Job
Women 52% Men 48%	≥20 years old 3% ≥30 years old 7% ≥40 years old 7% ≥50 years old 35% ≥60 years old 31% ≥70 years old 17%	Living alone 52% Divorced 14% Widowed 14% Married/cohabiting 20%	Health workers 10% Freelance 7% Teacher 14% Private practice 14% Unemployed 24% Retired 31%

## Data Availability

Data sharing not applicable.

## Supplementary Materials

The following supporting information can be downloaded at: https://www.mdpi.com/article/10.3390/ani13182968/s1, Description form on hoarders.

## References

[1] American Psychiatric Association (2002). Diagnostic and Statistical Manual of Mental Disorders (DSM).

[2] Barlow D.H., Durand V.M., Hofmann S.G. (2015). Abnormal Psychology: An Integrative Approach.

[3] Pinto de Sousa Miguela S.R., Ligabue-Braunb R. (2019). Compulsive hoarding as an atavism. Med. Hypotheses.

[4] Williams B. (2014). Animal hoarding—Recognition and possible interventions. Practice.

[5] Gahr M., Connemann B.J., Freudenmann R.W., Kolle M.A., Schonfeldt-Lecuona C.J. (2014). Animal hoarding: A mental disorder with implications for public health. Fortschritte Neurol. Psychiatr..

[6] Bratiotis C., Schmalisch C.S., Steketee G. (2011). The Hoarding Handbook: A Guide for Human Service Professionals.

[7] American Psychiatric Association (2014). Manual Diagnóstico e Estatístico de Transtornos Mentais (DSM-5).

[8] Paloski L.H., Ferreira E.A., Costa D.B., del Huerto M.L., de Oliveira C.R., de Lima Argimon I.I., Irigaray T.Q. (2017). Transtorno de acumulação de animais: Uma revisão sistemática. Psico.

[9] Joffe M., O’Shannessy D., Dhand N.K., Westman M., Fawcett A. (2014). Characteristics of persons convicted for offences relating to animal hoarding in New South Wales. Aust. Vet. J..

[10] Ockenden E.M., De Groef B., Marston L. (2015). Animal Hoarding in Victoria, Australia: An Exploratory Study. Anthrozoos.

[11] Calvo P., Duarte S., Bowen J., Bulbena A., Fatjó Y.J. (2014). Characteristics of 24 cases of animal hoarding in Spain. Anim. Welf..

[12] Cunha G.R., Martins C.M., Ceccon-Valente M.F., Silva L.L., Martins F.D., Floeter D., Robertson J.V., Ferreira F., Biondo A.W. (2017). Frequency and spatial distribution of animal and object hoarder behavior in Curitiba, Parana State, Brazil. Cad. Saude Publica.

[13] Marijana V., Dimitrijevic I. (2007). Body condition and physical care scales in three cases of dog hoarding from Belgrade. Acta Vet..

[14] Wilkinson J., Schoultz M., King H.M., Neave N., Bailey C. (2022). Animal hoarding cases in England: Implications for public health services. Front. Public Health.

[15] Apostol L., Rebega O., Miclea M. (2013). Psychological and Socio-demographic Predictors of Attitudes toward Animals. Procedia Soc..

[16] Berry C., Patronek G., Lockwood R. (2005). Long-term outcomes in animal hoarding cases. Anim. Law.

[17] Leon A.F., Sanchez J.A., Romero M.H. (2020). Association between Attitude and Empathy with the Quality of Human-Livestock Interactions. Animals.

[18] Stumpf B.P., Calacio B., Branco B.C., Wilnes B., Soier G., Soares L., Diamante L., Cappi C., Lima M.O., Rocha F.L. (2023). Animal Hoarding: A systematic review. Braz. J. Psychiatry.

[19] d’Angelo D., Ciani F., Zaccherini A., Tafuri S., Avallone L., d’Ingeo S., Quaranta A. (2020). Human-Animal Relationship Dysfunction: A Case Study of Animal Hoarding in Italy. Animals.

[20] Ascione F.R. (2010). International Handbook of Animal Abuse and Cruelty: Theory, Research, and Application (New Directions in the Human-Animal Bond).

[21] Nadal Z., Ferrari M., Lora J., Revollo A., Nicolas F., Astegiano S., Díaz Videla M. (2020). Noah’s syndrome: Systematic review of animal hoarding disorder. Hum. Anim. Interact. Bull..

[22] Mathes B.M., Morabito D.M., Schmidt N.B. (2019). Epidemiological and Clinical Gender Differences in OCD. Curr. Psychiatry Rep..

[23] Patronek G.J. (1999). Hoarding of animals: An under-recognized public health problem in a difficult-to-study population. Public Health Rep..

[24] Frost R.O., Patronek G., Rosenfield E. (2011). Comparison of object and animal hoarding. Depress. Anxiety.

[25] d’Angelo D., Chirico A., Sacchettino L., Manunta F., Martucci M., Cestaro A., Avallone L., Giordano A., Ciani F. (2021). Human-Dog Relationship during the First COVID-19 Lockdown in Italy. Animals.

[26] Ogata N., Weng H.Y., Messam L.L.M. (2023). Temporal patterns of owner-pet relationship, stress, and loneliness during the COVID-19 pandemic, and the effect of pet ownership on mental health: A longitudinal survey. PLoS ONE.

[27] Steketee G., Gibson A., Frost R.O., Alabiso J., Arluke A., Patronek G. (2011). Characteristics and antecedents of people who hoard animals: An exploratory comparative interview study. Rev. Gen. Psychol..

[28] Hoarding of Animals Research Consortium (HARC) (2002). Health implications of animal hoarding. Health Soc. Work.

[29] Lockwood R. (2018). Animal hoarding: The challenge for mental health, law enforcement, and animal welfare professionals. Behav. Sci. Law.

[30] Arluke A., Sanders C. (2009). Between the Species: Readings in Human-Animal Relations.

[31] Brakoulias V., Milicevic D. (2015). Assessment and treatment of hoarding disorder. Australas. Psychiatry.

[32] Topal J., Miklosi A., Csanyi V., Doka A. (1998). Attachment behavior in dogs (Canis familiaris): A new application of Ainsworth’s (1969) Strange Situation Test. J. Comp. Psychol..

[33] Herzog H. (2010). Some We Love, Some We Hate, Some We Eat: Why It’s So Hard to Think Straight about Animals.

[34] Herzog H. (2014). Biology, culture, and the origins of pet-keeping. Anim. Cogn..

[35] d’Angelo D., Sacchettino L., Carpentieri R., Avallone L., Gatta C., Napolitano F. (2022). An Interdisciplinary Approach for Compulsive Behavior in Dogs: A Case Report. Front. Vet. Sci..

[36] Sacchettino L., Gatta C., Maruccio L., Boncompagni C., Napolitano F., Avallone L., d’Angelo D. (2023). Combining cannabis and melatonin treatment with a rehabilitation program improved symptoms in a dog with compulsive disorder: A case report. Res. Vet. Sci..

[37] d’Angelo D., Sacchettino L., Quaranta A., Visone M., Avallone L., Gatta C., Napolitano F. (2022). The Potential Impact of a Dog Training Program on the Animal Adoptions in an Italian Shelter. Animals.

[38] Sacchettino L., Gatta C., Chirico A., Avallone L., Napolitano F., d’Angelo D. (2023). Puppies Raised during the COVID-19 Lockdown Showed Fearful and Aggressive Behaviors in Adulthood: An Italian Survey. Vet. Sci..

[39] Patronek G., Nathanson J.N. (2016). Understanding Animal Neglect and Hoarding, in Animal Maltreatment: Forensic Mental Health Issues and Evaluations.

[40] Avery L. (2005). From helping to hoarding to hurting: When the acts of “good Samaritans” become felony animal cruelty. Valpso. Univ. Law Rev..

[41] Reinisch A.I. (2008). Understanding the human aspects of animal hoarding. Can. Vet. J..

