# Supplementary material for: Description of Twenty-Nine Animal Hoarding Cases in Italy: The Impact on Animal Welfare
Source: Animals (Basel). 2023 Sep 20;13(18):2968. doi: 10.3390/ani13182968 (PMC10525848; doi:10.3390/ani13182968)
Supplement: Supplementary file 1 [file animals-13-02968-s001.zip › animals-2550237-supplementary.pdf]

Description form on hoarders

1. The origin of the complaint:

☐ Stranger	☐ Friend or acquaintance	☐ Non-living relative
☐ Family member or<br>☐ roommate	☐ Agency for social services	☐ Service person visiting a<br>☐ house
☐ Owner/tenant/neighbors	☐ Fire brigades or<br>☐ police department	☐ Veterinarian
☐ Anonymous/ passer-by	Altro	

2. People living in the home (age, sex, and relationship with the hoarder):

person	age	gender		Type of relationship with te hoarder
hoarder		O M	O F	
hoarder		O M	O F	
hoarder		O M	O F	
hoarder		O M	O F	

3. Marital status of the hoarder:

O Single	O married	O divorced	O widow/or	O cohabit/companion	O unknown

4. Residence type:

O single house	O Apartment/condominium	O Other (describe)

5. Job of the hoarder: ____________________

6. Other stakeholders involved in the case:

Department of Mental Health	O YES	O NO	O Not detected
Fire department	O YES	O NO	O Not detected
Local police	O YES	O NO	O Not detected
Foresters	O YES	O NO	O Not detected
Social assistants	O YES	O NO	O Not detected
Department of Child Protection	O YES	O NO	O Not detected
Tribunal	O YES	O NO	O Not detected
Other (indicate)	O YES	O NO	O Not detected

7. Degree of environmental neglect:

Area of the house	degree of disorder				
Bedroom	O None	O moderate	Oevident	O severe	O Not detected
Bathroom	O None	O moderate	Oevident	O severe	O Not detected
Living room	O None	O moderate	Oevident	O severe	O Not detected
Kitchen	O None	O moderate	Oevident	O severe	O Not detected
Dining room	O None	O moderate	Oevident	O severe	O Not detected
Stairs	O None	O moderate	Oevident	O severe	O Not detected
Corridor	O None	O moderate	Oevident	O severe	O Not detected
Under roof	O None	O moderate	Oevident	O severe	O Not detected
Outdoor area	O None	O moderate	Oevident	O severe	O Not detected
Basement	O None	O moderate	Oevident	O severe	O Not detected
Garage	O None	O moderate	Oevident	O severe	O Not detected

8. The following appliances/utilities were functional:

Stove/heaters	○ YES ○ NO ○ Not detected	Refrigerator/freezer	○ YES ○ NO ○ Not detected
Oven	○ YES ○ NO ○ Not detected	Bathroom sink	○ YES ○ NO ○ Not detected
Sink	○ YES ○ NO ○ Not detected	Toilet	○ YES ○ NO ○ Not detected
Electricity	○ YES ○ NO ○ Not detected	Water heater	○ YES ○ NO ○ Not detected
Washing machine	○ YES○ NO ○ Not detected	Shower/bath	○ YES ○ NO ○ Not detected

9. List number, type, clinical and behavioural signs of all accumulated animals:

Kind of animal	Number of animals in altered clinical and behavioral conditions	Number of animals seriously ill,injured or extremely malnourished	Number of deaths	Total
Dogs				
Cats				
Birds				
Reptiles				
Small mammals(describe)				
Horses				
Sheep/goats				
NOTES				

10. Motivation provided by the hoarder:

☐ Save the animals
☐ Love for animals
☐ Animals as children
☐ Nobody else wanted to take care of animals
☐ Animals are the only friend or companion of the hoarder.

11. Case resolution:

a. All the animals were taken away (seizure)
b. Some animals were taken away (partial seizure)
c. The accumulator has been banned from owning animals for a period
d. The accumulator has agreed to continue to be monitored
e. The accumulator has been subjected to psychiatric examination or mandatory medical treatment
f. The accumulator has been placed in a rehabilitation structure

g. The accumulator has been submitted to legal procedure.
